# Qualitative Assessment of Early Adverse Effects of Pfizer–BioNTech and Sinopharm COVID-19 Vaccines by Telephone Interviews

**DOI:** 10.3390/vaccines9090950

**Published:** 2021-08-26

**Authors:** Sami Abu-Halaweh, Rami Alqassieh, Aiman Suleiman, Mohammed Qussay Al-Sabbagh, Maram AbuHalaweh, Duaa AlKhader, Rozan Abu-Nejem, Roa’a Nabulsi, Mohammad Al-Tamimi, Mallak Alwreikat, Mazen Alnouti, Bayan Suleiman, Moh’d Yousef, Mohammad El Jarbeh, Abdel-Ellah Al-Shudifat, Ahmad Alqassieh, Isam Bsisu

**Affiliations:** 1 Department of Anesthesia and Intensive Care, School of Medicine, The University of Jordan, Amman 11942, Jordan; s.halaweh@ju.edu.jo (S.A.-H.); Mazen.alnouti@yahoo.com (M.A.); Bayan.suleiman93@gmail.com (B.S.); Yousefmohammad1993a@gmail.com (M.Y.); Mhmd.sum@hotmail.com (M.E.J.); 2 Department of General and Specialized Surgery, Faculty of Medicine, Hashemite University, Zarqa 13133, Jordan; Rami_qaisieh@yahoo.com; 3 Beth Israel Deaconess Medical Center, Anesthesia and Intensive Care Department, Harvard Medical School, Boston, MA 02215, USA; Asuleima@bidmc.harvard.edu; 4 Department of Neurology, University of Kansas Medical Center, Kansas City, KS 66160, USA; mqs.sabbagh@gmail.com; 5 Department of Internal Medicine, School of Medicine, The University of Jordan, Amman 11942, Jordan; maram.halaweh@gmail.com; 6 Department of Surgery, Medical University of South Carolina, Charleston, SC 29425, USA; do3a2_alkader@yahoo.com; 7 Faculty of Medicine, Hashemite University, Zarqa 13133, Jordan; Rozannijem.rn@gmail.com (R.A.-N.); Roaa.nabulsi@hotmail.com (R.N.); mohammad.altamimi@hu.edu.jo (M.A.-T.); mawreikat@gmail.com (M.A.); amalmufleh@hotmail.com (A.-E.A.-S.); 8 Division of Transplant Surgery, Department of Surgery, Medical University of South Carolina, Charleston, SC 29425, USA; ahmadsq1988@yahoo.com

**Keywords:** COVID-19, pandemic, vaccine, Pfizer–BioNTech, Sinopharm

## Abstract

Vaccines are considered the best approach for countering the COVID-19 pandemic. In this study, we compared early side effects associated with vaccination with the Sinopharm and Pfizer–BioNTech COVID-19 vaccines. Participants of this observational cohort were interviewed based on semi-structured telephone interviews, with enquiries about side effects that developed after vaccination with each dose of these vaccines. Overall, 1004 participants were enrolled, of which 51.1% received Sinopharm vaccine and 48.9% received the Pfizer–BioNTech vaccine. After the first dose, 46.3% of participants had an adverse reaction, with injection site pain most commonly being reported (33.2%). Participants who received the Pfizer–BioNTech vaccine had significantly higher frequencies of all types of adverse reactions (*p* < 0.01), with no significant differences in the duration of adverse reactions between the two vaccines. Regarding the second dose, 48.6% of participants had adverse reactions, with injection site pain being most commonly reported (29%). Those who received the Pfizer vaccine reported higher frequencies of all adverse reactions (*p* < 0.01). However, a longer duration of adverse reactions was seen among Sinopharm vaccine recipients as compared to Pfizer–BioNTech vaccine recipients (*p* = 0.01). In conclusion, early adverse effects are reported following all types of vaccines but these are more likely to be encountered following the administration of new-generation vaccines. These side effects are mostly mild and treatable.

## 1. Introduction

Vaccines have been proven to be the best approach for countering viral pandemics throughout human history [1]. The most recent viral pandemic is the ongoing COVID-19 pandemic, with the first cases reported in Wuhan, China, in December 2019 [2]. The pandemic triggered a global vaccine production race. At the end of 2020, 259 COVID-19 vaccine projects were ongoing worldwide, with 11 in phase III clinical trials [3].

Vaccines are classified according to their technological platforms. The most common type of classification categorizes vaccines as either classical or new generation [4]. Classical vaccines use either live-attenuated viruses, inactivated viruses, or virus-like particles and protein subunits, while new-generation vaccines implement nucleic acids, viral vectors, or antigen-presenting cells [5,6]. Of the COVID-19 vaccines that have completed phase III trials, the classical vaccine Sinopharm, produced by China National Pharmaceutical Group in China, and the new-generation vaccine Pfizer–BioNTech, produced by Pfizer in the United States, were the first available, and are the most widely distributed vaccines worldwide [5,7,8]. To date, no vaccine can be labelled as entirely free of side effects, but most of these side effects are either preventable or treatable [9]. In some vaccines, early side effects related to immune surge, such as fever, pain, myalgias and headaches, have been reported in phase I and II trials [10,11].

In this study, we aim to compare early side effects associated with vaccination with classical and new-generation vaccines, represented by the Sinopharm and Pfizer-BioNTech vaccines, respectively. This study is of importance time- and knowledge-wise as it provides a better insight into early adverse effects after the vaccination of larger populations.

## 2. Materials and Methods

### 2.1. Study Design

This observational cohort was conducted prospectively between 10 March 2021 and 2 April 2021, during which 1004 participants were enrolled in the study. This investigation included Jordanian adults with no history of previous allergies who had been vaccinated with either the Sinopharm or Pfizer–BioNTech COVID-19 vaccines. All included patients were vaccinated with the 2 doses of the vaccine after registering their demographic information, history of medical illnesses, and vaccination preferences using the online platform provided by the Jordanian Ministry of Health [12]. Patients who received Oxford/AstraZeneca, Sputnik V, or Moderna vaccines were not included in this cohort. It is worth mentioning that by 7 April 2021 (after the completion of data collection), only 119,621 individuals out of 10.1 million Jordanians (1.196%) had received complete vaccination with the 2 required doses of the vaccines (https://ourworldindata.org/covid-vaccinations?country=JOR; accessed on 10 April 2020). Therefore, using the aforementioned numbers and a sample size of 1004 individuals with a 95% confidence interval, the calculated margin of error was 0.67%. Hence, there is a 95% chance that the real value is within ±0.67% of the surveyed value.

### 2.2. Data Collection

Participants were interviewed based on semi-structured interviews by medical students. The interviews were conducted by calling the patients using their registered mobile phone numbers. Participants consented verbally to enrolment in the study after receiving information on study objectives and the expected duration for the interview. We first inquired about demographic data, past medical illnesses, history of COVID-19, and the type of the administered COVID-19 vaccine. We then queried the potential side effects that developed after vaccination with each dose of the vaccine. The main side effects inquired about were general weakness, headache, muscle pain, fever, chills, rigors, arthralgia, swelling or pain at site of injection, abdominal pain, nausea, flu-like symptoms, skin rash, and allergic reactions. We also documented the duration from vaccination to the development of these adverse medical events.

### 2.3. Ethical Approval

The study protocol was approved by the institutional review board (IRB) committee at Hashemite University (No. 6/7/2020/2021). No personal information was included in the interview. All participants were granted the right to withdraw from the study without the need to provide a reason for their request. The entries were coded using the national numbers of the participants as deidentifiers, and the collected data were then used solely for statistical analysis.

### 2.4. Statistical Analysis

Statistical analysis was performed using STATA (Stata Statistical Software: Release 16. College Station, TX, USA: StataCorp LLC). Group differences in the baseline characteristics in terms of the used vaccine were assessed using the chi-squared test. Thereafter, differences on prevalence of adverse reactions between each vaccine group were assessed by chi-squared test. Student’s *t*-test as well as the Mann–Whitney U test were used to assess for group differences in terms of time of onset and duration of the symptoms, depending on the normality of data distribution as assessed by the Shapiro–Wilk test. For convenience, all continuous variables were presented as mean ± standard deviation regardless of the normality of the data. A Kaplan–Meier failure curve was used to compare adverse reactions improvement time between the 2 vaccine groups.

## 3. Results

### 3.1. Characteristics of the Sample

Table 1 shows the baseline characteristics for the 1004 participants. The participants were divided into two groups based on the COVID-19 vaccine received, with 51.1% participants receiving two doses of the Sinopharm vaccine and 48.9% receiving two doses of the Pfizer–BioNTech vaccine. Most of the participants were male (67%), aged above 70 years (52.3%), and were classified as overweight (48.7%). Regarding chronic illnesses, 42.6%, 30%, 11.3%, and 7.6% had hypertension, diabetes, dyslipidemia, and coronary artery diseases, respectively. Only 4% of the sample has been infected previously with COVID-19, and around 33.1% were active smokers. The type of the vaccine differed significantly based on age (*p* < 0.01) and the presence of hypertension or coronary artery diseases (*p* < 0.01).

### 3.2. Frequency of Adverse Reactions Based on the Vaccine

**Figure 1:**
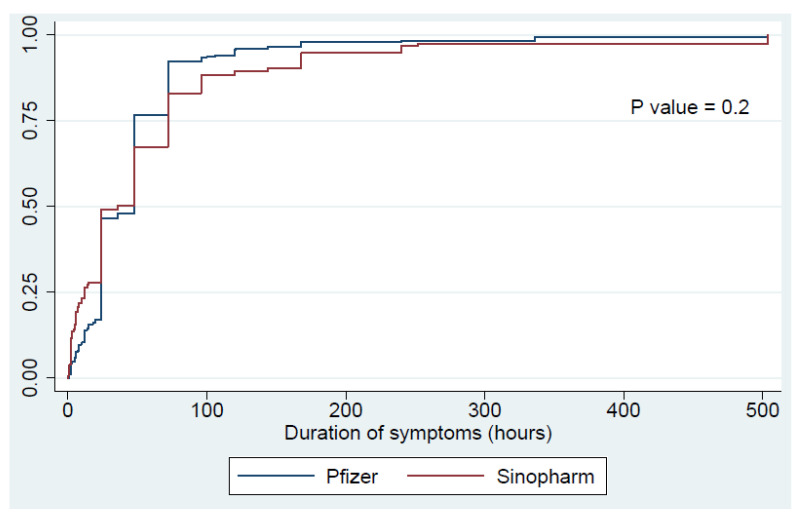
Improvement time of the adverse reactions following the first dose of the vaccine.

**Figure 2:**
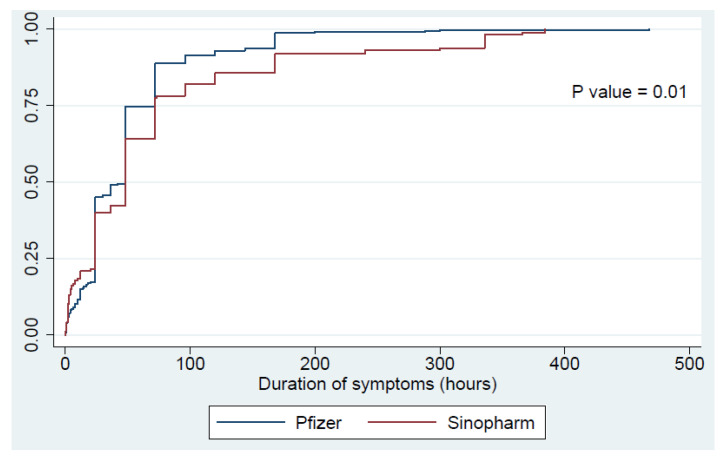
Improvement time of the adverse reactions following the second dose of the vaccine.

Table 2 presents the frequency of adverse reactions based on the vaccine. After the first dose of the vaccine, 46.3% of the participants had an adverse reaction regardless of the type of the vaccine. More specifically, 33.7% had local adverse reactions while 23.7% had systemic adverse reactions. Overall, participants who received the Pfizer–BioNTech vaccine had a significantly higher frequency of all types of adverse reactions (*p* < 0.01). Figure 1 presents a time analysis of the improvement rates of these adverse reactions, showing no significant differences in duration of adverse reactions between the two types of vaccination. For the second dose, 48.6% of the participants had adverse reactions, with 30% and 31.2% having local and systemic reactions, respectively. Again, those who were administered the Pfizer–BioNTech vaccine had higher frequencies of all adverse reactions (*p* < 0.01). Interestingly, Figure 2 shows a significantly longer duration of adverse reactions among participants who received the second dose of Sinopharm vaccine as compared to those who received the Pfizer–BioNTech vaccine (*p* = 0.01).

**Figure 3:**
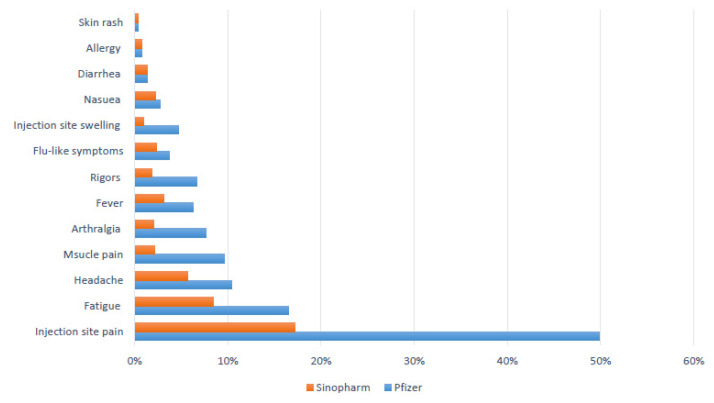
The frequency of adverse reactions after the first dose of COVID 19 vaccine.

Regarding the specific adverse reactions for the first dose, the most common described adverse reactions were injection site pain (33.2%), fatigue (12.4), headache (8%), muscle pain (5.8%), arthralgia (4.8%), fever (4.7%), and rigors (4.2%). All these reactions were significantly more frequent with the Pfizer–BioNTech vaccine group (*p* < 0.01). The frequencies, times of onset, as well as durations for individual adverse reactions are presented in Table 3 and Figure 3.

**Figure 4:**
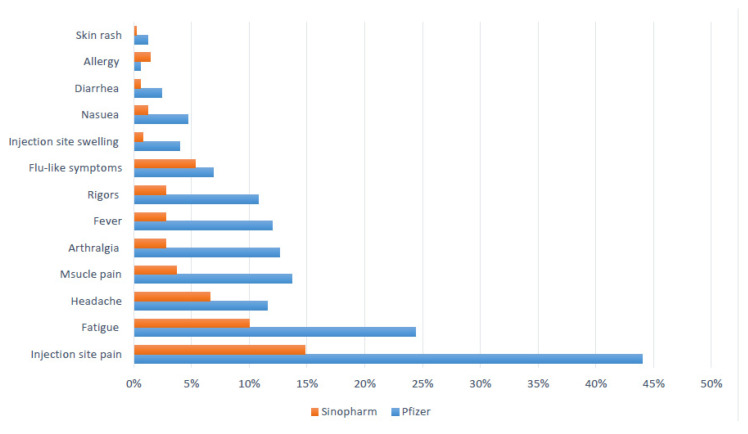
The frequency of adverse reactions after the second dose of the COVID 19 vaccine.

For the second dose, the most common reported adverse reactions were injection site pain (29%), fatigue (17%), headache (9.1%), muscle pain (8.6%), arthralgia (8.4%), fever (7.3%), and rigors (6.7%). All these reactions were significantly more frequent in the Pfizer–BioNTech vaccine group (*p* < 0.01). The frequencies, times of onset, and durations for individual adverse reactions are presented in Table 4 and Figure 4.

## 4. Discussion

The adverse effects of vaccines can be categorized into local and systemic effects, with varying degrees of severity [13]. Adverse reactions to COVID-19 vaccines were reported in early randomized clinical trials conducted to assess their efficacy [14]. While many review studies have compared different classes of COVID-19 vaccines on a scientific basis, few comparative studies have been conducted with real-time population samples [15,16,17,18].

The Pfizer–BioNTech vaccine is a messenger ribonucleic acid (mRNA) vaccine which encodes for the severe acute respiratory syndrome coronavirus 2 (SARS-CoV-2) spike protein [19,20]. Phase I and II clinical trials revealed elevated levels of RBD-specific IgG antibodies with a mean concentration 8 to 46.3 times the titer of a convalescent serum [19]. Sinopharm is a classical inactivated viral vaccine, with early animal studies suggesting that the vaccine confers protection without Ab-dependent enhancement [21].

Approximately 46.3% of participants had at least one adverse reaction regardless of the type of vaccine given after the first dose. Of those, 62.5% received the Pfizer–BioNTech vaccine, while 30.8% were administered the Sinopharm vaccine. The most common reported adverse effects after first and second doses were pain at injection site, followed by fatigue, headaches, and myalgia. All these effects were significantly greater in the Pfizer–BioNTech group. During phase I trials of the Pfizer–BioNTech vaccine, participants reported mild to moderate adverse reactions. Overall, 50% and 8% of the reported adverse events were associated with the vaccine or a placebo, respectively [19]. The most common reported adverse reaction was pain at the site of injection, which was more common after the second dose. This matches our findings where the most common reported adverse effect after the first and second dose was pain at injection site. In phase II trials, mild to moderate-intensity pain at the site of injection was the most common reported adverse reaction, with less frequency of pain among patients above the age of 55, and less frequency after the second dose. In a phase I trial, 83% reported adverse reactions after the first dose as compared to 14% with placebo, and 78% after the second dose as compared to 12% with placebo in participants aged 16 to 55. In total, 71% reported adverse reactions after administration of the first dose of the vaccine as compared to 9% of placebo recipients, and 66% reported adverse events after the second dose compared to 8% with placebo in participants above 55 [22]. In a phase I trial of the Sinopharm vaccine, 46% reported adverse reactions in the vaccine group compared to 38% in the placebo group, and the percentage was much lower in participants above 60 in the vaccine group (4%) [23]. Pain at the site of injection was the most common reported adverse reaction in participants above and below 60 years of age, with frequencies of 4% and 17%, respectively [23].

For systemic reactions, the most reported reactions in our study were fatigue, headaches and myalgias. In total, 48.6% of participants had adverse reactions following the second dose, with significantly higher frequencies for all types of reactions in patients who received the Pfizer–BioNTech vaccine. Nevertheless, patients receiving the second dose of the Sinopharm vaccine had a significantly longer duration of adverse reactions. Tremor, which is one of the most distressing systemic adverse effects, was reported more frequently after the second dose. In a phase II trial of the Pfizer–BioNTech vaccine, fatigue was reported in 59% of vaccine recipients and 23% of those in the placebo group aged 16–55, but these figures dropped to 39% and 17% in the vaccine and placebo group, respectively, in participants aged over 65 [22]. Headaches were reported in 52% of vaccine recipients, and less than 2% of vaccine recipients reported severe systemic reactions [22]. In a phase I trial of Sinopharm, fewer patients reported systemic adverse effects in the vaccine group (4%) as compared to placebo group (6%) [22]. The most common adverse reactions reported were fatigue (3%), inappetence (1%), and headaches (1%) [16,23].

The aim of conducting this study was to compare the side effects of the two available vaccines in Jordan. Studies reporting side effects can greatly counter the rumors that fuel the hesitancy of societies towards vaccination [24]. Many countries have established common access portals to monitor the safety of COVID-19 vaccines. In the United States, the Vaccine Adverse Event Reporting System is a governmental surveillance system that was established during the initial phase of national vaccination program [25]. In Jordan, an interactive governmental surveillance portal has been established to report side effects, but no awareness campaigns have been developed to teach participants how to use it [12]. Even in the present study, the side effects were based on self-reported side effects during structured telephone interviews. Hence, the described findings can be considered subjective, since the nature, intensity and duration of these adverse events could have been influenced by several individual-dependent variables, such as sociodemographic factors, mood, sensitivity to symptoms, and medical awareness.

Few studies have been conducted in Jordan to assess awareness, hesitancy, and side effects regarding COVID-19 vaccines. In a study conducted on health care workers in Jordan who received the AstraZeneca Vaxzevria, Pfizer-BioNTech, and Sinopharm vaccines [26], researchers found that 74% of participants reported pain at the site of injection, while the most common systemic adverse reactions reported were fatigue (52%), myalgia (44%), headaches (42%), and fever (35%). In this study, no serious adverse reactions were reported, and frequencies of adverse reactions were within the known common range for these vaccines.

The main limitation of this study is that it is an observational study based on semi-structured interviews via telephone calls. Although this allowed us to reach a broader group of patients during the study´s timeframe (during the second peak of the pandemic in Jordan), it limited our ability to investigate the severity of the adverse events post vaccination. The reporting of the severity of these adverse effects might be subjective, and a standardized method is required for evaluation in future studies. Moreover, the lack of a proper governmental system that follows serious adverse events and the lack of proper funding for this study to follow patients on a physical basis limited our ability to evaluate ICU-hospitalized patients and those who had dramatic adverse events, such as permanent and grave injuries or death. Hence, we recommend the activation of a governmental follow-up system to avoid such limitations in the future. In addition, post-vaccination antibodies titers, the efficacy of the vaccines, and long-term side effects must be vigorously investigated in future investigations.

## 5. Conclusions

In conclusion, the most common early adverse effect after COVID-19 vaccination is pain at site of injection. Early adverse effects are reported following all types of vaccines but are more likely to be encountered following the administration of new-generation vaccines. These side effects are mostly mild and treatable. An adverse event reporting system for vaccines is highly encouraged as a governmental surveillance system to provide accurate data regarding these adverse reactions.

## Figures and Tables

**Table 1:** Baseline characteristics of the 1004 vaccinated individuals, in terms of the used vaccine.

Variable	Categories	Total	Pfizer	Sinopharm	*p*-Value
**Total**		1004 (100)	491 (48.9)	513 (51.1)	
**Gender**					0.7
	Male	673 (67)	332 (49.3)	341 (50.7)	
	Female	331 (33)	159 (48)	172 (52)	
**Age**					<0.01
	18–30	13 (1.3)	5 (38.5)	8 (61.5)	
	31–40	36 (3.6)	22 (61.1)	14 (38.9)	
	41–50	262 (26.1)	160 (61)	102 (39)	
	51–60	118 (11.7)	30 (25.4)	88 (74.6)	
	61–70	50 (5)	31 (62)	19 (38)	
	above 70	525 (52.3)	243 (46.3)	282 (53.7)	
**BMI**					0.08
	Underweight	10 (1)	8 (80)	2 (20)	
	Normal	259 (25.8)	116 (44.8)	143 (55.2)	
	Overweight	488 (48.7)	249 (51)	239 (49)	
	Obese	246 (24.5)	117 (47.6)	129 (52.4)	
**Chronic diseases**					
	Hypertension	428 (42.6)	186 (43.5)	242 (56.5)	<0.01
	Coronary artery disease	76 (7.6)	26 (34.2)	50 (65.8)	<0.01
	Diabetes	301 (30)	149 (49.5)	152 (50.5)	0.8
	Thyroid disorders	36 (3.6)	16 (44.4)	20 (55.6)	0.6
	Dyslipidemia	113 (11.3)	49 (43.4)	64 (56.6)	0.2
	Respiratory disorder	21 (2)	9 (42.9)	12 (57.1)	0.6
**Currently smoking**		332 (33.1)	176 (53)	156 (47)	0.07
**History of COVID-19 infection**		41 (4)	17 (41.5)	24 (58.5)	0.3

Values are represented as number (percent).

**Table 2:** Frequency and type of adverse reactions in terms of the used vaccine.

First Dose of the Vaccine					Second Dose of the Vaccine				
Adverse Reaction	Total	Pfizer	Sinopharm	*p*-Value	Adverse Reaction	Total	Pfizer	Sinopharm	*p*-Value
Having an adverse reaction	465 (46.3)	307 (62.5)	158 (30.8)	<0.01	Having an adverse reaction	488 (48.6)	320 (65.2)	168 (32.8)	<0.01
Local side effects	338 (33.7)	247 (50.3)	91 (17.7)	<0.01	Local side effects	293 (30)	217 (44.2)	76 (14.8)	<0.01
Systemic side effects	238 (23.7)	148 (30.2)	90 (17.5)	<0.01	Systemic side effects	313 (31.2)	200 (40.7)	113 (22)	<0.01

Values are represented as number (percent).

**Table 3:** The frequencies, onset, and duration of the specific adverse reactions after the first dose of the vaccine.

	Frequency				Onset of Symptoms (Hours)				Duration of Symptoms (Hours)			
Adverse Reaction	Total	Pfizer	Sinopharm	*p*-Value	Total	Pfizer	Sinopharm	*p*-Value	Total	Pfizer	Sinopharm	*p*-Value
**Local adverse reactions**												
Pain at the site of the injection	333 (33.2)	245 (49.9)	88 (17.2)	<0.01	8.9 ± 10.4	9.4 ± 9.9	7.8 ± 11.4	0.2	39.4 ± 34.5	40.4 ± 35.3	36.5 ± 32.1	0.4
Swelling at site of injection	28 (2.8)	23 (4.7)	5 (1)	<0.01	20.5 ± 20.4	18.5 ± 18	29 ± 29.8	0.3	51.4 ± 37.7	47.9 ± 30.8	67.2 ± 62.1	0.3
**Systemic adverse reaction**												
General weakness	124 (12.4)	81 (16.5)	43 (8.4)	<0.01	18.3 ± 21	17.4 ± 17	20.1 ± 21	0.4	58 ± 84	43.3 ± 43.9	83.9 ± 124.6	0.01
Headache	80 (8)	51 (10.4)	29 (5.7)	<0.01	20 ± 34.3	11.4 ± 11	35.4 ± 52.5	<0.01	38.1 ± 34.3	34.4 ± 28.7	45.5 ± 43.1	0.2
Muscle pain	58 (5.8)	47 (9.6)	11 (2.1)	<0.01	23.2 ± 32.8	21.3 ± 27.9	30.5 ± 48.6	0.4	55 ± 81	45.4 ± 52.4	91.9 ± 144	0.09
Rigors	42 (4.2)	33 (6.7)	9 (1.8)	<0.01	22.3 ± 30.7	20.4 ± 31.5	28.9 ± 28.3	0.5	42.3 ± 81.8	48.5 ± 91	21.2 ± 21.3	0.4
Arthralgia	48 (4.8)	38 (7.7)	10 (2)	<0.01	36.3 ± 70	33.4 ± 21.3	46.1 ± 70	0.4	44.7 ± 83.6	27 ± 28	103 ± 157.6	0.01
Fever	47 (4.7)	31 (6.3)	16 (3.1)	0.02	19.8 ± 26	17.8 ± 26.6	23.3 ± 25.4	0.5	34.5 ± 37.1	32 ± 32.8	30.3 ± 45.3	0.5
Abdominal pain/diarrhea	14 (1.4)	7 (1.4)	7 (1.4)	0.9	40.7 ± 45.5	28.3 ± 24.7	28.8 ± 64.4	0.3	31.9 ± 30.9	40.8 ± 28.9	25.5 ± 32.8	0.4
Nausea/vomiting	24 (2.4)	13 (2.7)	11 (2.2)	0.6	35.2 ± 54.4	27.3 ± 42.7	43.1 ± 65.1	0.5	34.4 ± 28.5	31.9 ± 19.8	37.2 ± 36.8	0.7
Skin rash	4 (0.4)	2 (0.4)	2 (0.4)	0.9	92.3 ± 162.8	180 ± 220.6	4.5 ± 2.1	0.4	13 ± 15.6	13 ± 15.6	13 ± 15.6	1
Allergic reaction	8 (0.8)	4 (0.8)	4 (0.8)	1	7 ± 16.6	40 ± 13.9	16 ± 9.8	0.04	145.5 ± 158	75 ± 62.3	216 ± 203.7	0.2
Flu-like symptoms	30 (3)	18 (3.7)	12 (2.3)	0.2	53.3 ± 75.2	55.6 ± 82.9	40.1 ± 62.4	0.8	92.8 ± 102.1	72.6 ± 109	127.7 ± 82	0.2

Values are represented as numbers (percentages) and mean ± standard deviation.

**Table 4:** The frequencies, onset, and duration of the specific adverse reactions after the second dose of the vaccine.

	Frequency				Onset of Symptoms (Hours)				Duration of Symptoms (Hours)			
Adverse Reaction	Total	Pfizer	Sinopharm	Adverse Reaction	Total	Pfizer	Sinopharm	Adverse Reaction	Total	Pfizer	Sinopharm	Adverse Reaction
**Local Adverse reactions**												
Pain at the site of the injection	292 (29)	216 (44)	76 (14.8)	<0.01	9.9 ± 14.8	10.3 ± 12.1	8.7 ± 20.3	0.4	46.5 ± 60.8	44 ± 46	46.5 ± 60.8	0.7
Swelling at site of injection	24 (2.4)	20 (4)	4 (0.8)	<0.01	11.4 ± 12.2	10.7 ± 12.5	14.1 ± 11.8	0.6	50.5 ± 30.6	49.2 ± 31.3	57 ± 9	0.7
**Systemic Adverse reactions**												
General weakness	171 (17)	120 (24.4)	51 (10)	<0.01	19.5 ± 26.5	17.1 ± 15.6	25.5 ± 42.3	0.06	54.1 ± 61.8	45.5 ± 36.4	75.1 ± 97.2	<0.01
Headache	91 (9.1)	57 (11.6)	34 (6.6)	<0.01	21.2 ± 21.1	18.5 ± 29.3	25.6 ± 21.9	0.1	51.3 ± 46.1	45.7 ± 40.2	61 ± 54	0.1
Muscle pain	86 (8.6)	67 (13.7)	19 (3.7)	<0.01	24.8 ± 25	20.7 ± 21.1	39 ± 32.3	<0.01	58 ± 60.8	45 ± 32.1	103.6 ± 103.6	<0.01
Rigors	67 (6.7)	53 (10.8)	14 (2.8)	<0.01	27.1 ± 41.8	22.7 ± 30.5	43.4 ± 68.7	0.1	43.2 ± 52.6	38.9 ± 35.7	59.1 ± 92	0.2
Arthralgia	84 (8.4)	62 (12.6)	22 (4.3)	<0.01	64.5 ± 110.5	38.4 ± 32.4	136.3 ± 192.9	<0.01	52.4 ± 87.8	45.9 ± 82.7	69.7 ± 100	0.3
Fever	73 (7.3)	59 (12)	14 (2.8)	<0.01	18 ± 17.5	16.9 ± 14.8	22.4 ± 26.2	0.3	50.7 ± 61	44.8 ± 38.8	75.3 ± 113.6	0.09
Abdominal pain/diarrhea	15 (1.5)	12 (2.4)	3 (0.6)	0.02	35.9 ± 38.5	28.9 ± 27.3	64 ± 69.3	0.2	42.7 ± 41.7	42.4 ± 45.8	44 ± 25	0.96
Nausea/vomiting	29 (2.9)	23 (4.7)	6 (1.2)	<0.01	29.9 ± 45	30.9 ± 45.5	26 ± 46.8	0.8	29 ± 42.4	46 ± 44.1	18.1 ± 27.9	0.15
Skin rash	7 (0.7)	6 (1.2)	1 (0.2)	0.05	17.6 ± 15.1	21.3 ± 20.2	3	N/A	34.5 ± 68.9	2	63 ± 62	N/A
Allergic reaction	10 (1)	3 (0.6)	7 (1.4)	0.2	32.6 ± 20	34.7 ± 33.3	18 ± 9.3	0.3	109.3 ± 93.8	56 ± 13.8	136 ± 107	0.3
Flu-like symptoms	61 (6.1)	34 (6.9)	27 (5.3)	0.3	50.9 ± 59.6	30.1 ± 43.3	77 ± 67.3	<0.01	79.3 ± 70.6	12 ± 70.3	80.8 ± 72.2	0.9

Values are represented as numbers (percentages) and mean ± standard deviation. N/A: not applicable.

## Data Availability

The data presented in this study are available on request from the corresponding author.
